# A unique de novo gain-of-function variant in *CAMK4* associated with intellectual disability and hyperkinetic movement disorder

**DOI:** 10.1101/mcs.a003293

**Published:** 2018-12

**Authors:** Michael Zech, Daniel D. Lam, Sandrina Weber, Riccardo Berutti, Kamila Poláková, Petra Havránková, Anna Fečíková, Tim M. Strom, Evžen Růžička, Robert Jech, Juliane Winkelmann

**Affiliations:** 1 Institut für Neurogenomik, Helmholtz Zentrum München, Munich, 85764, Germany;; 2 Klinik und Poliklinik für Neurologie, Klinikum rechts der Isar, Technische Universität München, Munich, 81675, Germany;; 3 Institut für Humangenetik, Helmholtz Zentrum München, Munich, 85764, Germany;; 4 Department of Neurology and Center of Clinical Neuroscience, First Faculty of Medicine, Charles University and General Faculty Hospital, Prague, 120 00, Czech Republic;; 5 Institut für Humangenetik, Technische Universität München, Munich, 81675, Germany;; 6 Lehrstuhl für Neurogenetik, Technische Universität München, Munich, 80333, Germany;; 7 Munich Cluster for Systems Neurology, SyNergy, Munich, 81377, Germany

**Keywords:** athetoid cerebral palsy, language impairment, motor deterioration, psychomotor deterioration, torticollis

## Abstract

Calcium/calmodulin-dependent protein kinases (CaMKs) are key mediators of calcium signaling and underpin neuronal health. Although widely studied, the contribution of CaMKs to Mendelian disease is rather enigmatic. Here, we describe an unusual neurodevelopmental phenotype, characterized by milestone delay, intellectual disability, autism, ataxia, and mixed hyperkinetic movement disorder including severe generalized dystonia, in a proband who remained etiologically undiagnosed despite exhaustive testing. We performed trio whole-exome sequencing to identify a de novo essential splice-site variant (c.981+1G>A) in *CAMK4*, encoding CaMKIV. Through in silico evaluation and cDNA analyses, we demonstrated that c.981+1G>A alters *CAMK4* pre-mRNA processing and results in a stable mRNA transcript containing a 77-nt out-of-frame deletion and a premature termination codon within the last exon. The expected protein, p.Lys303Serfs*28, exhibits selective loss of the carboxy-terminal regulatory domain of CaMKIV and bears striking structural resemblance to previously reported synthetic mutants that confer constitutive CaMKIV activity. Biochemical studies in proband-derived cells confirmed an activating effect of c.981+1G>A and indicated that variant-induced excessive CaMKIV signaling is sensitive to pharmacological manipulation. Additionally, we found that variants predicted to cause selective depletion of CaMKIV's regulatory domain are unobserved in diverse catalogs of human variation, thus revealing that c.981+1G>A is a unique molecular event. We propose that our proband's phenotype is explainable by a dominant *CAMK4* splice-disrupting mutation that acts through a gain-of-function mechanism. Our findings highlight the importance of *CAMK4* in human neurodevelopment, provide a foundation for future clinical research of *CAMK4*, and suggest the CaMKIV signaling pathway as a potential drug target in neurological disease.

## INTRODUCTION

Calcium (Ca^2+^) signaling is of vital importance to human physiology and plays diversified roles in biological and pathological cerebral functions (Rosenberg and Spitzer 2011; Bading 2013). Within the Ca^2+^-driven biochemical machinery, the Ca^2+^/calmodulin-dependent serine/threonine protein kinases (CaMKs) are a class of master signaling effectors critically involved in various stages of neural development, including proliferation, migration, differentiation, and survival (Penzes et al. 2008; Takemoto-Kimura et al. 2017). In the mammalian brain, there are at least five known CaMKs: CaMKI, CaMKII, CaMKIII, CaMKIV, and CaMK kinase (CaMKK) (Swulius and Waxham 2008). Among these, CaMKIV is widely expressed in telencephalon and cerebellum, where it is predominantly localized to cell nuclei and orchestrates synapse-to-nucleus communication via determination of transcriptional outcomes (Jensen et al. 1991; Bading 2013). Human CaMKIV, encoded by *CAMK4* on Chromosome 5q22.1, is a monomeric 473-amino acid enzyme and subdivided into two main functional domains (Tokumitsu et al. 1997; Anderson et al. 2004; Naz et al. 2016): a protein kinase domain at the amino terminus (residues 46–300) and an autoregulatory domain at the carboxyl terminus (residues 305–341). The autoregulatory domain contains an autoinhibitory domain (AID) (residues 305–321), immediately followed by a calmodulin-binding domain (CBD) (residues 322–341), and forms a binding site for protein phosphatase 2A (PP2A). Because of its crucial involvement in Ca^2+^-dependent neuronal integrity, CaMKIV catalytic function is tightly controlled. In particular, unregulated autonomous activity of CaMKIV is prevented by molecular mechanisms involving its autoregulatory domain: (1) in the absence of Ca^2+^ stimulation, the AID blocks the catalytic core of the kinase domain, thereby maintaining CaMKIV in an autoinhibited state; (2) for release of autoinhibition, CaMKIV must bind an active Ca^2+^/calmodulin complex, requiring proper interaction with the CBD; and (3) following activation, CaMKIV is subject to rapid enzymatic deactivation, mediated by an association of the autoregulatory sequence with PP2A (Tokumitsu et al. 1997; Anderson et al. 2004; Naz et al. 2016).

Although CaMKs have attracted widespread research interest, the only indication that these enzymes are connected to human brain disease comes from the very recent identification of heterozygous dominant mutations in *CAMK2A*/*CAMK2B* (the genes encoding subunits of CaMKII) in individuals with nonsyndromic intellectual disability (Küry et al. 2017). Whether perturbation of other members of the CaMK family also causes Mendelian disease was still unknown. In this article, we report a second CaMK-related neurodevelopmental disorder. We describe a proband presenting with an unusual phenotype comprising intellectual disability and progression to severe movement disorder, who was found to harbor a germline de novo essential splice-site variant in *CAMK4*. We demonstrate that the splicing alteration results in heterozygous expression of CaMKIV with a truncated autoregulatory domain, mimicking in vitro–engineered deletion mutants that have been used for decades in the study of constitutive CaMKIV activation.

## RESULTS

### Clinical Presentation and Diagnostic Investigation

**Figure 1.**
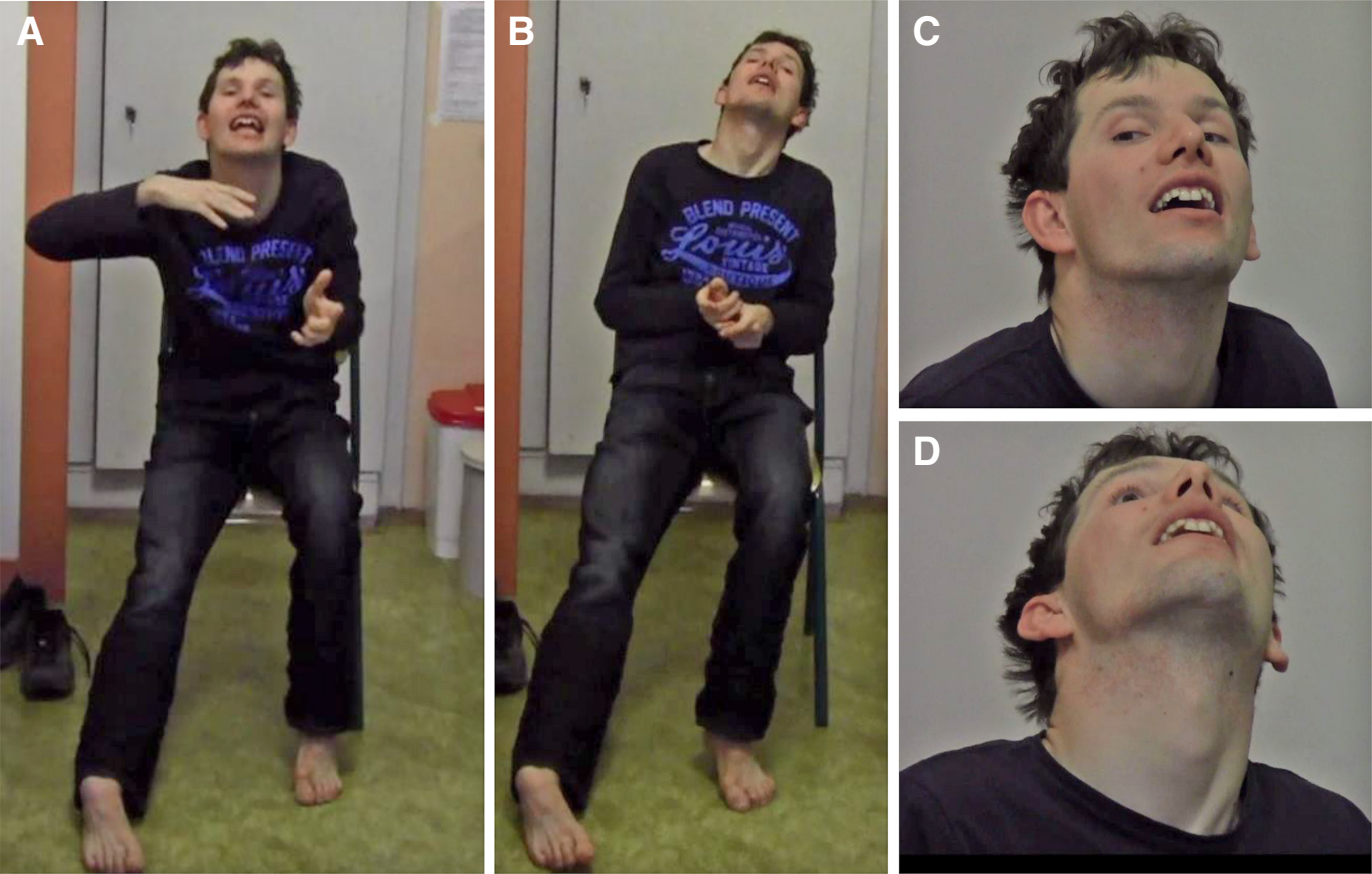
Pictures of proband A-4 with a de novo variant in *CAMK4.* Photographs of proband A-4 at the age of 28 yr showing his whole body (*A*,*B*) and close-ups of his face (*C*,*D*). Note involuntary abnormal postures.

**Figure 2.**
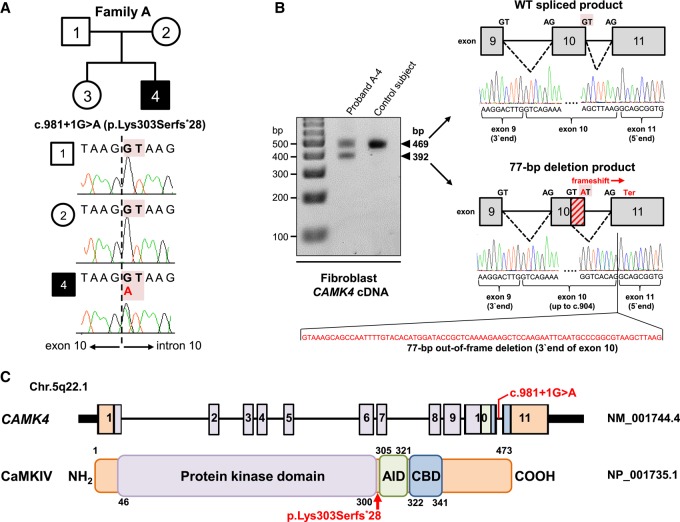
Identification of a de novo heterozygous essential splice-site variant, c.981+1G>A, in *CAMK4* and its predicted consequences at the transcript and protein level. (*A*) Pedigree for the investigated family A and *CAMK4* splice-disrupting variant in genomic DNA. The affected individual (proband A-4) is shaded black. Sanger sequencing confirmed the de novo status of the exome-identified c.981+1G>A variant located within the canonical GT splice donor of the penultimate exon 10 of *CAMK4*. (*B*) RT-PCR followed by gel electrophoresis and Sanger analysis in dermal fibroblasts from proband A-4 and a control subject. Apart from the 469-bp normal size cDNA product, proband A-4's cells produce, in equal amount, a second product of higher mobility (392 bp). Direct sequencing revealed that the 392-bp product is the result of utilization of a cryptic GT splice site upstream of the mutated donor, with generation of an abnormal mRNA isoform lacking the final 77 nt of exon 10 (diagonally hashed rectangle), leading to a frameshift and the introduction of a premature termination codon in the last exon of *CAMK4* (p.Lys303Serfs*28). WT, wild-type. (*C*) Diagram (not drawn to exact scale) of the human *CAMK4* locus (NM_001744.4) and its encoded protein (NP_001735.1). The identified c.981+1G>A variant is shown relative to exonic structure and the position of the protein truncation (p.Lys303Serfs*28) is highlighted with a red arrow. Numbering refers to exons (*upper* panel) and amino acids (*lower* panel). Key protein domains are illustrated in color: purple for the protein kinase domain, green for the AID, and blue for the CBD.

We encountered a 28-yr-old male proband of European descent (proband A-4 in family A; see Fig. 2A) who was referred to the Department of Neurology and Center of Clinical Neuroscience at Charles University in Prague (Czech Republic) because of neurodevelopmental disability and generalized hyperkinetic movements (Table 1; Fig. 1). Prior to diagnostic and research investigations, written informed consent was obtained from the proband and his family members in accordance with standard protocols approved by local ethics review boards. Proband A-4 was the second child of healthy nonconsanguineous parents, aged 27 (mother, A-2 in Fig. 2A) and 29 yr (father, A-1 in Fig. 2A) at the time of conception. His older sister (A-3 in Fig. 2A) was healthy. There was no family history of neurological or neurodevelopmental disease. Although his mother suffered unspecific viral infection during pregnancy, he was born at full term via an uncomplicated vaginal delivery (normal Apgar score, birth weight and length > 50th percentile). Metabolic screening was unremarkable, and there were no congenital malformations. He experienced two episodes of transient cyanosis at age 6 wk with spontaneous resolution. Since the age of 3 yr, he has been under regular surveillance of medical experts because of global developmental delay. He was only able to sit on his own at 18 mo and failed to walk unassisted before the age 3 yr. He had motor coordination problems, with both coarse and fine movements being very clumsy. Speech development was profoundly impaired, such that by the age of 5 yr only a few short phrases were expressed. Over time, he was noted to have significant socialization difficulties and other behavioral abnormalities, including negativism, lack of emotional control, and autoaggressive reactions. Neuropsychological assessment at the age of 10 yr yielded a diagnosis of moderate intellectual disability and possible autism spectrum disorder. At this age, he was able to attend special education classes and speech therapy, producing some improvements in expressive language skills.

**Table 1.** Summary of genetic and clinical findings in this study

Gene information								
Gene	Chromosomal band	RefSeq canonical transcript	Genomic position (hg19)	Genomic size (bp)	Exon count	DOMINO^a^ score	pLI score	OMIM number
*CAMK4*	5q22.1	NM_001744.4	Chr 5:110,559,947-110,820,748	260,802	11	0.85 (very likely dominant)	0.02	114080

Variant information								
Genomic position (hg19)	Variation nucleotide	Variation amino acid	Variant type	Genotype/inheritance	CADD score^b^	Human Splicing Finder^c^ prediction	Frequency gnomAD	Frequency in-house exomes (*N* =12,000)
Chr 5:110,818,636	NM_001744.4: c.981+1G>A	NP_001735.1: p.Lys303Serfs*28	Essential splice-site	Heterozygous/ de novo dominant	26	Most probably affecting splicing	Not found	Not found

Phenotype information								
Age at most recent examination (years)	Gender	Descent	Family history	Congenital anomalies	Clinical features	Behavorial features	Brain MRI	Treatment attempts (unsuccessful)
28	Male	European (Czech)	Negative	None	Developmental delay, speech delay, intellectual disability, ataxia, dysarthria, mixed hyperkinetic movement disorder (generalized dystonia, choreoathetosis, myoclonus)	Sociability deficits, negativism, lack of emotional control, autoaggression, stereotypic behaviors	Cerebellar atrophy	Levodopa, anticholinergics, tiapride, botulinum toxin

pLI, probability of being loss of function intolerant, according to Lek et al. (2016); OMIM, Online Mendelian Inheritance in Man, https://omim.org/; CADD, Combined Annotation Dependent Depletion; gnomAD, Genome Aggregation Database, http://gnomad.broadinstitute.org/.

^a^DOMINO (https://wwwfbm.unil.ch/domino/) is an online tool assessing the probability of a given gene to be associated with autosomal dominant disease.

^b^Scores ≥ 20 indicate that a given variant is expected to be among the 1% most deleterious alterations in the human genome.

^c^Human Splicing Finder (http://umd.be/HSF3/) is an online tool assessing the effects of exonic and intronic variants on pre-mRNA splicing.

From the age of 13 yr onward, proband A-4's motor functions progressively deteriorated. He started to develop worsening limb coordination and difficulty walking, accompanied by the appearance of dystonic posturing of his left leg and forceful trunk retroflexion. In the beginning, involuntary movements were only occasionally observed, but within 12 mo, symptoms persisted throughout the day and had a tendency to spread. By the age of 15 yr, disabling twisting postures affected the neck, torso, and all four extremities. Additionally, he manifested irregular jerks and abnormal adventitious movements of the arms, more pronounced by intentional action, along with increasing articulation deficits. Neurological evaluation at the age of 16 yr revealed generalized dystonia (retro torticollis, dystonia of the trunk and limbs bilaterally) combined with choreoathetoid movements aggravated by voluntary actions, hyperkinetic dysarthria, myoclonus, and minor ataxic features. Physical examination was unremarkable and documented no gross dysmorphic stigmata. During a 12-yr follow-up, his neurologic disease course stabilized, despite some reports of intermittent worsening of baseline dystonia. On his most recent clinical examination at the age of 28 yr, proband A-4 continued to have a persistent mixed generalized hyperkinetic movement disorder, dominated by dystonia and chorea (Fig. 1). To some extent, hyperkinetic movements were superimposed with complex motor stereotypies. As a result of concomitant truncal and lower limb ataxia, he displayed unsteady gait but could walk without assistance.

Movement disorder symptoms were irresponsive to trialed medications including levodopa, anticholinergics, tiapride, and botulinum toxin. Magnetic resonance imaging (MRI) of the brain was notable for the presence of localized atrophy involving the cerebellum. Neither signs of leukodystrophy nor structural defects were evident in the basal ganglia, white matter, and corpus callosum. Proband A-4 had an extensive array of normal diagnostic evaluations that included electroencephalography, cerebrospinal fluid analysis, and nerve conduction studies, as well as numerous blood and/or urine biochemistry examinations (complete blood count, comprehensive metabolic panel, laboratory test for Wilson's disease, and profiling of amino acids, organic acids, triglycerides, and vitamins). Genetic testing composed of karyotyping, chromosomal microarray, and screening for DYT-*TOR1A*, DYT-*THAP1*, DYT-*GCH1*, spinocerebellar ataxias 1, 2, 3, 6, 7, 8, 12, 17, Friedreich's ataxia, and fragile X syndrome was negative.

To comprehensively delineate proband A-4's genetic profile and uncover potentially pathogenic DNA sequence variants, he was enrolled in a research study focusing on the etiologic origins of dystonia (Technical University of Munich and Helmholtz Center Munich). Proband–parent trio whole-exome sequencing (WES) was conducted, coupled with experimental characterization of the identified candidate causative variant.

### Genomic Analyses

For WES of proband A-4 and his biological parents, the average coverage depth across the exome-capture region was 161×, and 98.8%–99.1% of the target nucleotides were covered at a read depth of 20× or greater (Table 2). The trio WES data set was first studied in a search for pathogenic or likely pathogenic variations in published genes linked to neurodevelopmental disorders, intellectual disability, or heritable movement abnormalities, but no suspicious findings were revealed. Given proband A-4's severe clinical presentation and the fact that he was the only affected individual in his family, we next hypothesized that the syndrome was caused by either rare recessive variants or an ultra-rare damaging de novo dominant event in a single yet-undescribed disease-causing gene. Scrutiny of the WES data under the assumption of a recessive mode of inheritance for the observed clinical presentation detected four compound heterozygous variants in two genes, all of which were considered to have insufficient evidence for pathogenicity (for an explanation, see Supplemental Table). Bioinformatics subtraction analysis incorporating proband A-4's and both his parents’ WES variant profiles led to the identification of a single heterozygous de novo alteration, Chr 5: g.110818636G>A (c.981+1G>A [p.?]) at *CAMK4* (hg19, NM_001744.4), as an extremely interesting candidate for follow-up investigation (Tables 1 and 3). Evaluation of the full WES data sets demonstrated that *CAMK4* was the only gene that contained a high-confidence de novo variant call.

**Table 2.** Whole-exome sequencing parameters for proband A-4

Total number of reads	Total number of mapped reads	Mapped reads (%)	Mapped sequence (Gb)	Bases covered ≥20× (%)	Average exome coverage	Average read length (bp)	Coverage at *CAMK4* described variant	*CAMK4*, fraction at ≥20× coverage (%)
162,220,172	162,033,697	99.89	16.37	99.09	169.94×	101	67× (55% alternate allele)	100

**Table 3.** *CAMK4* variant characteristics

Gene	Chromosome	HGVS DNA reference	HGVS protein reference	Variant type	Predicted effect	dbSNP/dbVar ID	Genotype	ClinVar ID	Parent of origin
*CAMK4*	Chr 5: 110,818,636	NM_001744.4: c.981+1G>A	NP_001735.1: p.Lys303Serfs*28	Essential splice site	Aberrant splicing	Not available	Heterozygous	SCV000804199	De novo

**Figure 3.**
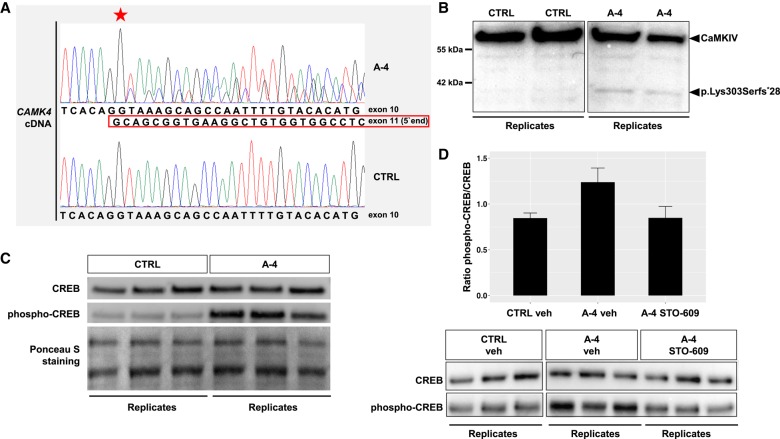
The *CAMK4* c.981+1G>A (p.Lys303Serfs*28) allele is expressed in proband-derived cells and engenders a gain-of-function effect. (*A*) Sanger chromatograms of fibroblast *CAMK4* cDNA from proband A-4 and a healthy control (CTRL). Proband A-4's cDNA shows wild-type and c.981+1G>A variant sequence. The nucleotide position at which the 77-bp deletion begins in proband A-4 is indicated with a star. (*B*) Western blot analysis using an anti-human CaMKIV antibody raised against the amino terminus of the protein. A truncated protein of ∼37 kDa (p.Lys303Serfs*28) was detected in proband A-4's cells (A-4) but not in control cells (CTRL). Boxes represent cropped sections from a digital image of a single membrane (an uncropped version of the image can be found in Supplemental Fig. S1). Two biological replicates are shown for each cell line (CTRL, A-4). (*C*) Western blot analysis of phospho-CREB and total CREB expression in fibroblasts from proband A-4 (A-4) and a healthy control (CTRL). Ponceau S staining was used as a loading control. Three biological replicates are shown for each cell line (CTRL, A-4). (*D*) (*Bottom*) Western blot analysis assessing phospho-CREB and total CREB expression in fibroblasts derived from proband A-4 and a healthy control (CTRL). Cells were treated with vehicle (veh) or STO-609, an inhibitor of CaMKK activity. Boxes represent cropped sections from digital images of the same membrane (uncropped versions of the images can be found in Supplemental Fig. S2). Three biological replicates are shown for each condition (CTRL veh, A-4 veh, A-4 STO-609). (*Top*) Bar plot showing results of densitometric quantification, depicting the ratio of phosphorylated CREB to total CREB. Data in the bar plot are presented as mean ± standard error of the mean, *n* = 3.

The prioritized *CAMK4* c.981+1G>A variant generated a GT>AT substitution in the ubiquitously conserved splice-donor dinucleotide of the penultimate exon 10 (Fig. 2A–C). The variant was unreferenced in the literature and dbSNP142 and not found in the 1000 Genomes and Genome Aggregation Database (gnomAD) data sets containing more than 248,000 population alleles. Moreover, the variant was unique in our own in-house exomes (*N* = 12,000) from individuals with a wide range of clinical diagnoses. Sanger sequencing of genomic DNA for proband A-4, his sister, and his parents proved that the c.981+1G>A variant had arisen de novo (Fig. 2A). Combined scores of CADD and splice-defect simulation software predicted that c.981+1G>A was in the top 1% of most deleterious mutations and likely to be functionally relevant because of a disruption of *CAMK4* pre-mRNA processing (Table 1; Desmet et al. 2009; Kircher et al. 2014). To directly test the latter prediction and evaluate the potential pathogenicity of the c.981+1G>A variant, we synthesized cDNA from isolated RNA of fibroblast cell lines of proband A-4 and a healthy control individual. PCR amplification of cDNA was performed with a primer set targeting *CAMK4* exon 7–8 junction (forward primer) and exon 11 (reverse primer). Whereas wild-type cells produced a single transcript with the expected PCR size (469 bp), proband A-4 expressed two products: the wild-type transcript and a smaller transcript of 392 bp (Fig. 2B). Sanger analysis of the proband-specific transcript revealed an aberrant mRNA species lacking the final 77 nt of the penultimate exon 10 because of the activation of a cryptic splice-donor site upstream of c.981+1G>A. This deletion altered the reading frame to introduce a premature termination codon in the last exon of *CAMK4*, leading to a predicted chain-truncated protein: p.Lys303Serfs*28 (Fig. 2B,C). Frameshift mutations that create premature termination codons within last exons are expected to render the resulting transcripts insensitive to nonsense-mediated decay (NMD) (Kervestin and Jacobson 2012; Lindeboom et al. 2016). Therefore, we reasoned that the c.981+1G>A *CAMK4* variant mRNA was not suppressed by NMD. In support of this conjecture, inspection of gel-separated RT-PCR amplicons revealed wild-type and aberrantly spliced *CAMK4* transcripts in similar proportion (Fig. 2B). Furthermore, sequencing of cDNA obtained from fibroblasts of proband A-4 showed the presence of both the wild-type and the truncated transcripts, although the latter were visualized at slightly lower levels (Fig. 3A). In total, our results strongly suggested that the c.981+1G>A allele led to stable heterozygous expression of an mRNA that was expected to translate into a product p.Lys303Serfs*28. The predicted consequence of the p.Lys303Serfs*28 variant is the production of CaMKIV protein with truncation of its autoregulatory domain, but preservation of its protein kinase domain (Fig. 2C).

### Cellular Protein Biochemical Studies

To examine the expression of *CAMK4* c.981+1G>A (p.Lys303Serfs*28) at the protein level, we performed western blot analysis in fibroblast cell lines obtained from proband A-4 and two healthy donors. In proband-derived cells, immunoblotting yielded a CaMKIV normal-size band and a second band of ∼37 kDa, corresponding to p.Lys303Serfs*28, which showed reactivity with an antibody against residues 131–147 of CaMKIV (Fig. 3B; Supplemental Fig. S1). Immunoblot band intensity indicated that the expression of p.Lys303Serfs*28 was lower than that of the full-length protein.

Numerous studies have shown that synthetic mutant CaMKIV species lacking the autoregulatory domain are constitutively active (Cruzalegui and Means 1993; Chatila et al. 1996; Anderson et al. 2004). Such constructs provide a powerful means of studying the consequences of up-regulated CaMKIV signaling in vitro and in vivo (Sun et al. 1994; Kane and Means 2000; See et al. 2001; Wu et al. 2002; Marie et al. 2005). To test the hypothesis that proband A-4's p.Lys303Serfs*28 variant also mediated enhanced activity, we assessed phosphorylation of cyclic AMP–responsive element-binding protein (CREB), a major downstream substrate of CaMKIV, in proband and control cells (Sun et al. 1994). Consistent with previously described observations in cells transfected with constitutively active CaMKIV mutants (Sun et al. 1994; Joseph and Turrigiano 2017), immunoblotting demonstrated a significant increase of CREB phosphorylation at serine residue 133 in proband-derived fibroblasts relative to controls (Fig. 3C). To confirm that this effect was mediated by CaMKIV, we treated cells with STO-609, a potent inhibitor of CaMKK, the upstream activator of CaMKIV (Tokumitsu et al. 2002). Notably, it has been demonstrated that CaMKIV lacking its autoregulatory domain still requires activation by CaMKK to adopt a constitutively active state (Chatila et al. 1996). As shown in Figure 3D (Supplemental Fig. S2), treatment of mutant cells with STO-609 restored phosphorylation of CREB at serine-133 to the basal control level. Collectively, our findings confirmed the production of a truncated CaMKIV protein in proband A-4 and provided experimental evidence for a c.981+1G>A (p.Lys303Serfs*28)-induced overactivation of CaMKIV signaling, which could be pharmacologically reversed by inhibition of CaMKK. We concluded that c.981+1G>A (p.Lys303Serfs*28) is a gain-of-function allele and that the effects of this variant are driven by the loss of CaMKIV's autoregulatory domain.

## DISCUSSION

A central principle of precision neurology is the search for personalized therapeutic interventions on the basis of causal gene identification, molecular pathway definition, and delineation of mutation-specific pathological mechanisms. Although the vast genetic heterogeneity of neurodevelopmental disorders and congenital movement disabilities has started to be appreciated (Deciphering Developmental Disorders 2015; Carecchio and Mencacci 2017; Lohmann and Klein 2017), we continue to encounter phenotypic presentations whose molecular underpinnings remain to be untangled. In the present study, we adopted an unbiased trio WES strategy to discover a de novo heterozygous *CAMK4* consensus splice-site alteration (c.981+1G>A) as the only suspicious finding in a proband who manifested a remarkable spectrum of neurodevelopmental deficits including global psychomotor delay, intellectual disability, autistic traits, adolescence-onset hyperkinetic movement disorder, ataxia, and cerebellar atrophy.

To understand the functional impact of the identified *CAMK4* splicing variant, we complemented our WES approach by predictive modeling, cDNA experimentation, and protein biochemical analysis. By doing so, we were able to show that the c.981+1G>A variant generated a truncated, stable mRNA coding for an autoregulatory domain-deficient version of CaMKIV (p.Lys303Serfs*28). Further, recapitulating the effects of engineered deletion of the CaMKIV autoregulatory element (Cruzalegui and Means 1993; Sun et al. 1994), proband-derived cells demonstrated an up-regulation of CaMKIV signaling, suggesting that c.981+1G>A (p.Lys303Serfs*28) confers the encoded protein with a gain of function. Finally, we generated evidence that the gain-of-function behavior of the c.981+1G>A (p.Lys303Serfs*28) allele was sensitive to specific inhibition of CaMKIV's physiological activator CaMKK.

Multiple lines of evidence support the de novo variant in *CAMK4* in this study as explanatory for the clinical syndrome of our proband. First, existing gene expression and mouse model data are consistent with a role for *CAMK4* in neurodevelopmental disease. According to publicly available data repositories (The Allen Mouse Brain Atlas, The Human Protein Atlas) (Lein et al. 2007; Uhlén et al. 2015) and previous studies (Jensen et al. 1991; Nakamura et al. 1995), CaMKIV is expressed with spatial and developmental temporal specificity, with significant enrichment in the brain as compared to other tissues. In the postnatal brain, CaMKIV is highly expressed in the granule and Purkinje cells of the cerebellum, which are crucial for coordination and motor control (Ohmstede et al. 1989). In addition, high levels of CaMKIV are present in hippocampal neurons mediating essential aspects of learning and memory (Bito et al. 1996). The functional significance of these expression patterns is reflected in mice harboring a genetic defect of *Camk4*. Ribar and colleagues produced *Camk4* null mice, which exhibited spontaneous tremulous and ataxic movements together with gross deficiencies in balance and sensorimotor performance (Ribar et al. 2000). The severe locomotor defects were accompanied by reduced numbers of Purkinje neurons, demonstrating that CaMKIV is required for normal development and function of the cerebellum (Ribar et al. 2000). Furthermore, Wei and colleagues, studying an independent null line, showed alterations in hippocampal physiology and memory function, thereby establishing a link between *Camk4* and cognitive development (Wei et al. 2002). Of note, in both studies, the modification of *Camk4* was toward homozygous loss of function. Nevertheless, these observations are in good agreement with the hypothesis that a mutation of CaMKIV contributed to the cognitive and motor symptoms in our proband.

**Figure 4.**
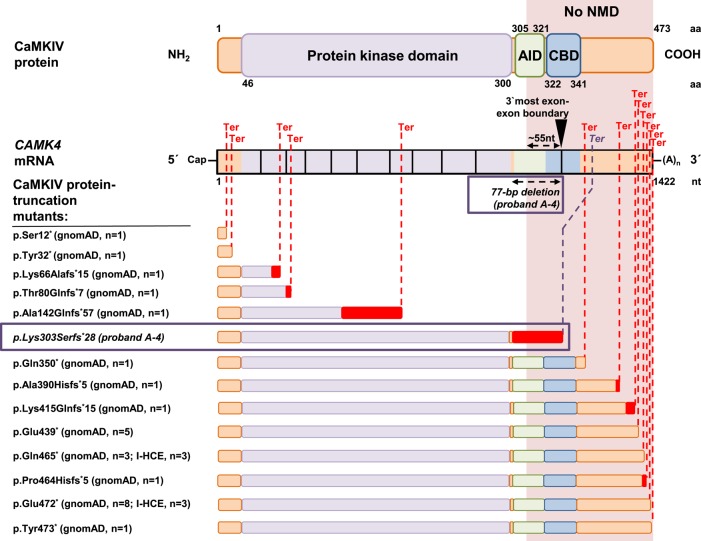
*CAMK4* variants predicting selective abrogation of the autoregulatory domain of CaMKIV are not found in human variation databases. *CAMK4* PTVs found in 123,136 control exomes of the Genome Aggregation Database (gnomAD), 12,000 in-house-sequenced control exomes (I-HCE), and proband A-4 are illustrated. Schematic representations of hypothetical and predicted expressed truncated proteins and locations of premature termination codons (Ter; dashed red lines) are depicted in relation to *CAMK4* mRNA and CaMKIV domain structures. The protein kinase domain is shown in purple, the autoregulatory domain is shown in green (AID) and blue (CBD), and frameshift sequence is colored red. Amino acid (aa) and nucleotide (nt) numbers are given. Also indicated is the last exon–exon boundary (exon10–exon11, black inverted triangle) along with the ∼55-nt position upstream of this boundary. PTVs producing termination codons beyond the last ∼55 nt of the penultimate exon are expected to result in truncated mRNAs that are resistant to NMD (no NMD; orange box extending through the figure). gnomAD documents 13 different PTVs (seven nonsense variants and six frameshift variants) in 26 independent exomes and our in-house exome data set two different PTVs (two nonsense variants) in six independent exomes. Of these PTVs, five are expected to cause truncation of all relevant protein domains (protein kinase domain plus autoregulatory domain) and trigger NMD. The remaining eight PTVs are expected to encode mRNAs that escape NMD but retain functionality of all relevant protein domains. Strikingly, the only exome-identified PTV that is predicted to (1) not trigger NMD and (2) cause selective abrogation of the autoregulatory domain of CaMKIV is proband A-4's p.Lys303Serfs*28 variant resulting from a 77-bp deletion in exon10 (boxed). Of note, gnomAD also contains two *CAMK4* essential splice-site variants (c.387-1G>A [p.?], c.459+2dupT [p.?]), both of which are predicted to cause out-of-frame skipping of exon 5 and thus deleteriously affect the protein kinase domain (not illustrated).

Second, the investigation of large genomic variation resources and gene-level probability scores indicates that the c.981+1G>A (p.Lys303Serfs*28) variant is a unique molecular event in a gene that is likely to be relevant to dominant non–haploinsufficient disease. Based on DOMINO, a tool for inferring the likelihood for a gene to carry dominant mutations, *CAMK4* is predicted to have a very high probability of producing human autosomal dominant clinical phenotypes (Table 1; Quinodoz et al. 2017). Curiously, interrogation of 123,136 control exomes of the gnomAD database and 12,000 in-house exomes from cohorts with unrelated diseases identifies several *CAMK4* protein-truncating variants (PTVs) that are expected to result in NMD of their mRNAs (Fig. 4), consistent with the prediction that *CAMK4* is tolerant to heterozygous loss-of-function variation (probability of being loss-of-function intolerant [pLI] score of 0.02, as estimated by Lek et al. [2016]). In contrast, c.981+1G>A (p.Lys303Serfs*28) is the only *CAMK4* PTV in our in-house exome collection predicted to encode an mRNA that evades NMD and results in a CaMKIV protein with selective depletion of its carboxy-terminal autoregulatory domain. Similarly, no such variant is registered in gnomAD (Fig. 4). Together, these findings buttress the hypothesis that *CAMK4* c.981+1G>A (p.Lys303Serfs*28) is a dominant disease-relevant mutation that causes our proband's phenotype via a pathophysiological mechanism other than heterozygous loss of protein activity. This interpretation is in accordance with recent studies demonstrating that rare carboxyl-terminal PTVs can be found in dominant disease genes and that these mutations do not act through haploinsufficiency (Simpson et al. 2011; Hood et al. 2012; White et al. 2015; Jansen et al. 2017; Palmer et al. 2017). We attempted to identify additional putative *CAMK4* gain-of-function variants (PTVs predicted to mediate selective loss of the autoregulatory domain of CaMKIV) in 100 internal exomes from Czech dystonia probands (Helmholtz Center Munich) and in more than 13,000 neurodevelopmental disease trio exomes available through denovo-db (Turner et al. 2017). However, no more mutations were found, implying that *CAMK4*-related clinical disease might be exceedingly rare.

Third, substantial amounts of available data suggest that enhancement of CaMKIV activity has detrimental neurophysiological effects. It is well known that CaMKIV is a pivotal mediator of brain activity–dependent Ca^2+^ dynamics in circuit development, neuronal transmission, and synaptic plasticity (Kang et al. 2001; Bading 2013). Via activation of transcription factors such as CREB, CaMKIV has long been implicated in the control of neuronal gene expression (Corcoran and Means 2001). Furthermore, a broad range of evidence has established CaMKIV as an important player in Ca^2+^-mediated regulation of alternative splicing (Xie and Black 2001; Xie et al. 2005). Considering these fundamental bioactivities, it is not surprising that deregulation of CaMKIV leads to disorganization of neuronal function. At the molecular level, constitutive activation of CaMKIV causes significant changes in transcription factor activities and might therefore compromise the coordinated expression of target gene programs required for brain development and neuronal homeostasis. As an example, Kane and Means have demonstrated that an activating CaMKIV mutation markedly alters transcriptional responses regulated by orphan nuclear receptors, which have well-recognized roles in the maturation of neuronal subtypes such as the cerebellar Purkinje cells (Kane and Means 2000). Interestingly, mutations of orphan nuclear receptor genes have been discovered to result in neurodevelopmental delay, intellectual disability, and cerebellar atrophy–associated motor anomalies, features also coincident with the *CAMK4* mutation identified here (Guissart et al. 2018). Increased CaMKIV activity also affects the fine-tuning of spatiotemporally controlled splicing patterns in neurons. Different studies have proven that overexpression of constitutively active CaMKIV results in an altered constitution of neuron-specific receptor and channel pre-mRNAs, including those encoding members of the NMDA-sensitive glutamate receptor family (Xie and Black 2001; Lee et al. 2007). CaMKIV overactivation could thereby disturb the electrical properties of neurons and distort long-term neuroadaptations. At the circuit level, up-regulation of the CaMKIV signaling cascade influences homeostatic mechanisms such as global synaptic scaling (Goold and Nicoll 2010). Recent work has shown that CaMKIV gain of function leads to substantial alterations in excitatory synaptic strength, which has been suggested to predispose to neuronal circuit disorders (Joseph and Turrigiano 2017). Of particular interest in this regard, the prevailing view holds hyperkinetic motor disease, as prominently evident in our proband, to be a consequence of circuit disorganization and impaired synaptic scaling (Calabresi et al. 2016; Jinnah and Hess 2018).

Integrating all these previous observations and our present data, we propose that the *CAMK4* variant we identified causes neuronal defects at both the molecular and circuit levels similar to those seen with engineered CaMKIV overactivation, which in turn might be the origin of the complex neurological phenotype presented by our proband.

In aggregate, we have described a neurological syndrome characterized by neurodevelopmental deficits evolving toward adolescence-onset hyperkinetic movement disorder. We uncovered a de novo gain-of-function variant in *CAMK4* as the likely driver of this condition, thereby documenting a key role for this gene in neurodevelopment in a human subject. Our finding that pharmacological manipulation normalized CaMKIV signaling in the presence of the identified gain-of-function variant highlights the possibility of a mechanism-directed treatment strategy. Although additional studies are required, we postulate that *CAMK4* gain-of-function mutation is an excellent candidate for neurodevelopmental disease causation and that therapeutic avenues addressing uncontrolled activation of CaMKIV in the brain should be explored further.

## METHODS

### Trio WES

Genomic DNA samples were extracted from venous blood lymphocytes of proband A-4 and his family members (parents A-1 and A-2, sister A-3) using conventional robotic techniques. Trio WES was performed on genomic DNA from proband A-4 and his parents. The full WES methodology, bioinformatic analysis, and variant interpretation protocols have been described before (Zech et al. 2016, 2017). In brief, exon targets were captured with the SureSelect All Exon system (Agilent Technologies) according to the manufacturer's recommendations. Barcoded exome libraries were pooled and sequenced using paired-end, 100-cycle chemistry on a HiSeq2500 high-throughput DNA sequencing machine (Illumina). After demultiplexing, sequences were aligned to the reference human genome (NCBI build 37, hg19 version) with the Burrows–Wheeler Aligner and variants were called with SAMtools, Pindel, and ExomeDepth.

### Variant Filtering and Prioritization

All called variants that did not pass high-confidence standards (read coverage < 10×, Phred-scaled quality score < 30) were filtered out. Variant calls were filtered further against in-house lists of known sequencing artifacts. WES data analysis was performed using a pipeline developed in-house (Helmholtz Center Munich). De novo alterations were extracted with custom scripts comparing WES data from the parents versus the proband, as previously reported (Zech et al. 2016). Of the variants called, candidates were prioritized according to the following criteria: (1) the frequency of the alternate allele (minor allele frequency < 0.001 [dominant variants] or < 0.005 [recessive variants]) in control exome data sets (1000 Genomes Project, dbSNP142, in-house exome database, and gnomAD); (2) the effect on amino acid sequence; (3) predicted pathogenicity; and (4) cosegregation with the phenotype. We only retained rare missense, nonsense, indel, stop-loss, and splice-related intronic variants that were classified as deleterious in at least one out of three in silico prediction algorithms (PolyPhen-2, SIFT, CADD) and matched allelic state based on autosomal dominant, autosomal recessive, and X-linked modes of inheritance. All selected variants were inspected manually using the Integrative Genomics Viewer (IGV) and cross-referenced with the Online Mendelian Inheritance in Man (OMIM, https://www.omim.org/) and ClinVar (https://www.ncbi.nlm.nih.gov/clinvar/) databases to check for publicly available clinical and/or functional information.

### Confirmation of the *CAMK4* Variant

The potential disease-associated variant detected through WES was verified via standard PCR amplification and Sanger sequencing. The following primer pair targeting the altered area in genomic DNA was used: forward: 5′-TCCGTGGGTCACAGGTAAAG-3′; and reverse: 5′-AGATGAAGAACGGTAGAACTATTATTG-3′.

### Complementary DNA (cDNA) Studies

We collected skin biopsies of proband A-4 and two healthy control individuals. Primary dermal fibroblasts were expanded as described earlier (Zech et al. 2015). Total fibroblast RNA was isolated by using the RNeasy kit (QIAGEN), and RNA integrity was determined using the Agilent 2100 Bioanalyzer with the use of RNA 6000 Nano chips. Reverse transcription was carried out via the SuperScript First-Strand Synthesis System for RT-PCR (Invitrogen) using 1000 ng of total RNA as a template. The primer pair for PCR of fibroblast-derived cDNA was designed to encompass *CAMK4* exons 9 and 10—forward: 5′-GGGTACTGCGCACCTGAAAT-3′ (in *CAMK4* exon 7–8 junction); and reverse: 5′-TTGGAGAAGGGTCTCGGCTA-3′ (in *CAMK4* exon 11). PCR products were electrophoretically separated on 2% agarose gels and visualized by Midori Green staining. Separated PCR products were extracted and purified from agarose gel using the QIAquick Gel Extraction Kit (QIAGEN). Sequence determination of cDNA amplicons was done by Sanger analysis using the cDNA primer set described above.

### Western Blot Analysis in Fibroblasts

Fibroblasts of proband A-4 and two healthy donors were maintained in Dulbecco's Modified Eagle Medium (DMEM) supplemented with 10% fetal bovine serum, 50 U/ml penicillin, and 50 g/ml streptomycin. Near-confluent fibroblasts were treated with STO-609 (5 µM; Merck) or vehicle (DMSO). Two hours later, lysates were prepared with lysis buffer (150 mM NaCl, 1% NP-40, 50 mM Tris, pH 8, protease inhibitors [cOmplete Mini, Merck]), followed by agitation and centrifugation. Supernatants were subjected to SDS-PAGE and semidry immunoblotting. Primary antibodies used (all rabbit, 1:1000) were anti-CaMKIV (ThermoFisher PA1-542), anti-CREB (Cell Signaling 9197), and anti-phospho-CREB (Cell Signaling 9198). Secondary antibody was HRP-conjugated donkey anti-rabbit IgG (BioLegend 406401). Chemiluminescent detection was performed with ECL Prime (Amersham). Protein abundance was quantified by signal density (ImageJ).

## ADDITIONAL INFORMATION

### Data Deposition and Access

Whole-exome sequencing data are not publicly available because consent could not be obtained. The *CAMK4* variant found in this study has been submitted to ClinVar (http://www.ncbi.nlm.nih.gov/clinvar/) under accession number SCV000804199.

### Ethics Statement

The study was approved by the local ethics review boards at Technical University of Munich, Germany, and Charles University in Prague, Czech Republic. Written informed consent was obtained from the proband and proband's parents for publication and accompanying images.

### Acknowledgments

We are grateful to the proband and his family for their participation in this study. M.Z. was supported by an internal research program at Helmholtz Center Munich, Germany (“Physician Scientists for Groundbreaking Projects”).

### Author Contributions

M.Z. and D.D.L. conceived the study, generated and analyzed the data, and drafted and critically reviewed the manuscript. S.W. generated and analyzed the data and critically reviewed the manuscript. R.B. and T.M.S. generated the data and critically reviewed the manuscript. K.P., P.H., A.F., and E.R. provided the clinical data and critically reviewed the manuscript. R.J. conceived the study, provided the clinical data, and critically reviewed the manuscript. J.W. conceived the study, analyzed the data, and drafted and critically reviewed the manuscript.

### Funding

This study was funded by the Czech Science Foundation (GACR16-13323S) as well as in-house institutional funding from Technische Universität München, Munich, Germany; Helmholtz Zentrum München, Munich, Germany, and Charles University, Prague, Czech Republic (PROGRES Q27). D.D.L. was supported by DFG grant LA 3830/1-1.

### Competing Interest Statement

The authors have declared no competing interest.

## Supplementary Material

Supplemental Material
