# Supplementary material for: A unique de novo gain-of-function variant in CAMK4 associated with intellectual disability and hyperkinetic movement disorder
Source: Cold Spring Harb Mol Case Stud. 2018 Dec;4(6):a003293. doi: 10.1101/mcs.a003293 (PMC6318768; doi:10.1101/mcs.a003293)
Supplement: Supplemental Material [file supp_4_6_a003293__index.html]

A unique de novo gain-of-function variant in CAMK4 associated with intellectual disability and hyperkinetic movement disorder — Supplemental Material 

# A unique de novo gain-of-function variant in *CAMK4* associated with intellectual disability and hyperkinetic movement disorder

## Supplemental Material

- Supplemental\_Figures\_Table.pdf
