# Supplementary material for: A unique de novo gain-of-function variant in CAMK4 associated with intellectual disability and hyperkinetic movement disorder
Source: Cold Spring Harb Mol Case Stud. 2018 Dec;4(6):a003293. doi: 10.1101/mcs.a003293 (PMC6318768; doi:10.1101/mcs.a003293)
Supplement: Supplemental Material [file supp_mcs.a003293_Supplemental_Figures_Table.pdf]

Supplemental Figure S1 Uncropped western blot from Figure 3B

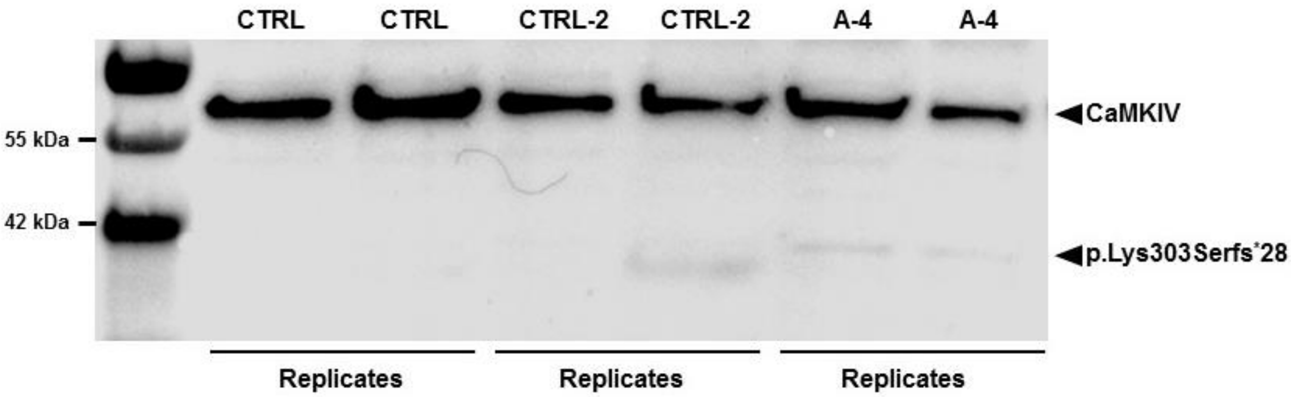

Western blot analysis using an anti-human CaMKIV antibody raised against the N-terminus of the protein. A truncated protein of ~37kDa (p.Lys303Serfs*28) was detected in proband A-4`s cells but not in control cells. The experiment was performed with fibroblasts derived from proband A-4 (A-4) and two healthy controls (CTRL, CTRL-2). Two biological replicates are shown for each cell line (CTRL, CTRL-2, A-4).

Supplemental Figure S2 Uncropped western blots and raw data from Figure 3D

Line	Treatment	Biological replicate	Ratio phospho-CREB/ CREB	Group mean	Group SEM
CTRL	Veh	1	0,921508064		
CTRL	Veh	2	0,879012188	0,845616429	0,0560038
CTRL	Veh	3	0,736329036		
CTRL	STO	1	0,737350621		
CTRL	STO	2	0,963836879	0,88704119	0,07485376
CTRL	STO	3	0,959936071		
A-4	Veh	1	1,315992206		
A-4	Veh	2	0,93983773	1,238676304	0,15510993
A-4	Veh	3	1,460198977		
A-4	STO	1	1,098995996		
A-4	STO	2	0,728294404	0,84822229	0,12542646
A-4	STO	3	0,71737647		

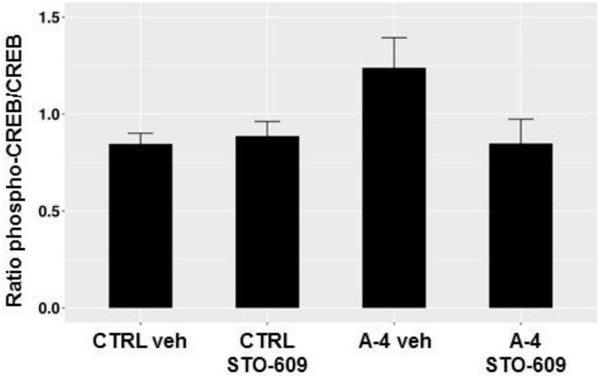

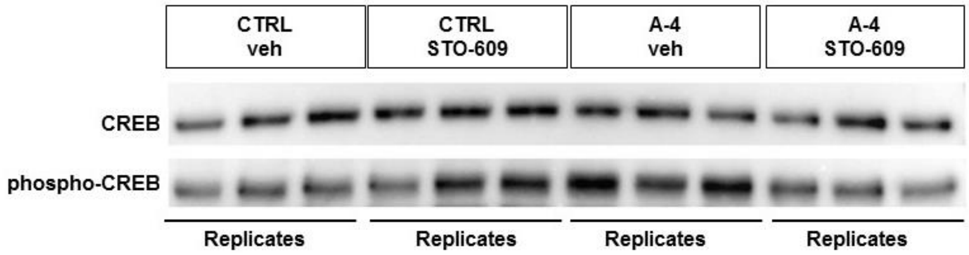

Bottom: western blot analysis assessing phospho-CREB and total CREB expression in fibroblasts derived from proband A-4 (A4) and a healthy control (CTRL). Cells were treated with vehicle (veh) or STO-609, an inhibitor of CaMKK activity. Three biological replicates are shown for each condition (CTRL-veh, CTRL-STO-609, A4-veh, A4-STO-609). Top: table and bar plot showing results of densitometric quantification, depicting the ratio of phosphorylated CREB to total CREB. Data in the bar plot are presented as mean ± standard error of the mean, n = 3.

Supplemental Table Recessive variants identified in proband A-4

Gene	OMIM disease-association	Genomic position (hg 19)	RefSeq transcipt	Variation nucleotide	Variation amino acid	Variant type	Known disease-causing mutation (ClinVar)	dbSNP142	Frequency in-house exomes (N=12,000) (allele count/ total allele number)	Frequency gnomAD (allele count/ total allele number)	Mode
EPPK1	no	chr8:144940706	NM_031308	c.6716G>A	p.Arg2239His	missense	no	rs112377501	98/24,000	1,035/219,198	compound heterozygous
EPPK1	no	chr8:144941879	NM_031308	c.5543C>T	p.Ala1848Val	missense	no	rs150969952	106/24,000	653/277,068	compound heterozygous
HYDIN	yes (MIM: 608647)	chr16:70934984	NM_001270974	c.8971C>A	p.Pro2991Thr	missense	no	not found	33/24,000	87/246,152	compound heterozygous
HYDIN	yes (MIM: 608647)	chr16:71212894	NM_001270974	c.318A>G	p.Ile106Met	missense	no	not found	not found	4/276,608	compound heterozygous

A search for rare biallelic variants in the trio-whole-exome sequencing datasets from proband A-4 and his parents detected four compound heterozygous variants in *EPPK1* and *HYDIN*. The *EPPK1* variants were deemed unlikely to be responsible for proband A-4`s neurodevelopmental phenotype because of the predominant role of *EPPK1* in keratinocyte proliferation and wound healing. In addition, we observed a large number (N=240) of in-house-sequenced exomes from individuals with no overlapping phenotypes that contained ≥2 rare non-synonymous variants in *EPPK1*. The *HYDIN* variants were dismissed because loss-of-function mutations in this gene cause primary ciliary dyskinesia type 5, characterized by progressive decline in lung function and recurrent airway infections, symptoms not seen in proband A-4. In all, an identifiable recessive locus for proband A-4`s clinical presentation was considered unlikely.
